# Intentional Weight Loss, Waist Circumference Reduction, and Mortality Risk Among Postmenopausal Women

**DOI:** 10.1001/jamanetworkopen.2025.0609

**Published:** 2025-03-06

**Authors:** Michael Hendryx, JoAnn E. Manson, Robert J. Ostfeld, Rowan T. Chlebowski, Erin S. LeBlanc, Molly E. Waring, Wendy E. Barrington, Marisa A. Bittoni, Sylvia Wassertheil-Smoller, Jackie Gofshteyn Herold, Juhua Luo

**Affiliations:** 1 School of Public Health, Indiana University Bloomington; 2 Department of Medicine, Brigham and Women’s Hospital, Harvard Medical School, Boston, Massachusetts; 3 Division of Cardiology, Montefiore Health System, Bronx, New York; 4 The Lundquist Institute, Torrance, California; 5 Permanente Center for Health Research, Portland, Oregon; 6 Department of Allied Health Sciences, University of Connecticut, Storrs; 7 Department of Epidemiology, School of Public Health, University of Washington, Seattle; 8 Division of Medical Oncology, The Ohio State University, Columbus; 9 Department of Epidemiology and Population Health, Albert Einstein College of Medicine, Bronx, New York; 10 Encoded Therapeutics, Irvington, New York; 11 Department of Epidemiology and Biostatistics, School of Public Health, Indiana University Bloomington

## Abstract

**Importance:**

Research investigating weight loss and mortality risk often fails to differentiate between intentional and unintentional weight loss and typically uses body mass index (BMI) as the measure of excess body weight.

**Objective:**

To evaluate associations between weight loss and waist circumference (WC) reduction and mortality, considering weight loss intentionality.

**Design, Setting, and Participants:**

This cohort study used data from the Women’s Health Initiative Observational Study, which had a prospective cohort with mean follow-up of 18.6 years ending in February 2023. The study included women aged 50 to 79 years at 40 clinical centers in the US. Women with missing data, cancer at baseline, or considered underweight at baseline were excluded. Data were collected from September 1993 to February 2023 and were analyzed from June to December 2024.

**Exposures:**

Measured weight loss and WC reduction between baseline and year 3, stratified by women who reported intentional weight loss or not.

**Main Outcomes and Measures:**

Outcomes included adjudicated all-cause, cancer, cardiovascular, and other mortality through the end of follow-up. Cox proportional hazards regression models were used to evaluate the associations (hazard ratios [HRs] and 95% CIs) between weight loss, WC reduction, and mortality over 18.6 years of follow-up.

**Results:**

This study included 58 961 women at baseline (mean [SD] age, 63.3 [7.2] years; mean [SD] BMI, 27.0 [5.6]; mean [SD] WC, 84.1 [13.0] cm). As of February 28, 2023, 29 183 women (49.5%) died from all causes. Intentional weight loss measured by questionnaire was associated with lower subsequent mortality rates for all-cause mortality (HR, 0.88; 95% CI, 0.86-0.90), cancer mortality (HR, 0.87; 95% CI, 0.82-0.92), cardiovascular mortality (HR, 0.87; 95% CI, 0.83-0.91), and other mortality (HR, 0.89; 95% CI, 0.86-0.92), comparing loss of 5 pounds or more to stable weight. Reported intentional weight loss coupled with actual weight reduction of 5% or more was associated only with lower cardiovascular mortality (HR, 0.90; 95% CI, 0.81-0.99). Reported intentional weight loss coupled with measured WC loss was associated with lower rates of all-cause mortality (HR, 0.91; 95% CI, 0.86-0.95), cancer mortality (HR, 0.85; 95% CI, 0.76-0.95), and cardiovascular mortality (HR, 0.79; 95% CI, 0.72-0.87). Unintentional weight loss or unintentional WC loss were each associated with increased mortality risk for all groups, as were weight gain and WC gain.

**Conclusions and Relevance:**

In this cohort study, reported intentional weight loss efforts that were coupled with measured WC reductions were associated with lower risk of all-cause, cancer, and cardiovascular mortality. Attention to diet and exercise that promote reductions in central adiposity should be encouraged.

## Key Points

**Question:**

Is intentional weight loss or waist circumference reduction associated with mortality risk in postmenopausal women?

**Findings:**

In this cohort study of 58 961 women aged 50 to 79 years at baseline, intentional efforts at weight reduction coupled with measured reduction in waist circumference was associated with significantly lower mortality risk over 18.6 years of follow-up for all-cause, cancer, and cardiovascular mortality. In contrast, measured intentional weight reduction alone was associated only with lower cardiovascular mortality risk.

**Meaning:**

These findings suggest that weight loss efforts for women should focus on lifestyle changes that will result in clinically meaningful reductions in visceral adiposity as measured by waist circumference.

## Teaser

This cohort study examines the association of intentional vs unintentional weight loss and waist circumference reduction and mortality among postmenopausal women in the US.

## Introduction

Globally, 2.6 billion people live with overweight or obesity.^1^ In the United States, the age-adjusted prevalence of obesity among adults is 42%, and it is 44% among women 60 years or older.^2^ The increasing prevalence of obesity is concerning because obesity raises the risk of type 2 diabetes, cardiovascular disease, certain cancers, reduces quality of life, and shortens life expectancy.^3^

Although the association between obesity and adverse health outcomes is well-established, evidence regarding the impact of weight loss on mortality remains inconsistent. Some studies report lower mortality risk among older adults who are overweight or obese compared with those with normal body mass index (BMI; calculated as weight in kilograms divided by height in meters squared)^4^ or suggest that weight loss may increase mortality risk.^5^ Media reports call this an obesity paradox and suggest to the public that it is healthier to remain overweight rather than to lose weight,^6^ generating uncertainty about the benefits of losing weight among older adults. However, studies about weight loss often have methodological limitations,^7,8,9^ including lack of data determining whether weight loss was intentional or unintentional, and relying on BMI as the sole measure of obesity.^10^

Unintentional weight loss can reflect underlying morbidity or adverse effects of treatment and, thus, can contribute to reverse causality in observational studies.^11,12,13,14^ Although weight loss efforts are encouraged in clinical settings and have been linked to health advantages, such as improved cardiovascular health,^15^ or lower obesity-related cancer incidence,^16^ whether intentional weight loss reduces mortality risk is not well understood. One review^17^ concluded that intentional weight loss among adults older than age 50 years was not associated with mortality, although this study did not address waist circumference (WC). A meta-analysis indicated that weight fluctuations increased mortality risk relative to stable weight but did not address WC and indicated that further research on intentionality was needed.^18^ In contrast, a meta-analysis of randomized clinical trials including adults of all ages indicated that intentional weight loss among adults with obesity significantly reduced mortality risk.^19^ However, knowledge regarding intentionality from prospective observational studies of populations in clinical settings rather than randomized clinical trials is limited, especially among older adults.^20,21^

Intentional weight loss may have different associations with health outcomes depending on whether weight loss results in reduction of central adiposity or reflects loss in muscle mass without loss in adiposity. Although BMI is a measure of body size, it does not measure abdominal adiposity. WC, which assesses abdominal adiposity, may be a stronger indicator of mortality risk than BMI, especially for older adults.^22,23,24,25^ Discrepancies between weight and WC likely reflect differences in muscle mass^26,27^; adults typically lose muscle mass and gain visceral adiposity as they age, which often results in a larger WC but an unchanged or lower BMI. Increases in WC may increase mortality risk,^21,28^ but results are inconsistent. Dai et al^20^ reported that WC is associated with cancer mortality but not other mortality causes among adults older than age 65 years, and O’Suilleabhain et al^29^ reported that WC was not an estimator of mortality outcomes among adults older than 80 years.

The uncertainty surrounding the benefits of intentional weight loss, and how it should be measured, obscure a potentially compelling public health message that older people with overweight or obesity could reduce their mortality risk by losing weight. To address this, we examined associations between changes in weight and in WC throughout a 3-year period corresponding to intentional vs unintentional efforts to lose weight and subsequent all-cause, cancer, and cardiovascular mortality during the next 18.6 years. We hypothesized that intentional weight loss efforts as indicated by reductions in weight or WC would each be associated with lower mortality. We also explored whether methods of weight loss efforts were associated with mortality.

## Methods

This cohort study followed the Strengthening the Reporting of Observational Studies in Epidemiology (STROBE) reporting guideline. It was approved by the institutional review boards at all a 40 clinical centers and at the coordinating center. All participants provided written informed consent.

### Women’s Health Initiative

We used data from the Observational Study (OS) of the Women’s Health Initiative (WHI), a large prospective cohort study.^30^ Details of the scientific rationale, eligibility requirements and baseline characteristics of WHI participants have been published elsewhere.^31,32^ A total of 161 808 women ages 50 to 79 years were recruited from 40 clinical centers throughout the US between September 1, 1993, and December 31, 1998. The WHI includes both clinical trial (CT) and observational study components. Participants in the observational study included 93 676 women who were screened for the CT but were ineligible or unwilling to participate or were recruited through a direct invitation to participate in the observational study. Women were followed annually for an average of 18.6 years after the year 3 visit.

### Study Population

The observational study sample initially included 93 676 women. We excluded 12 075 women who had a history of cancer (except nonmelanoma skin cancer) at baseline and 1194 who were underweight (BMI less than 18.5) at baseline, for a sample of 80 638. We also excluded 16 153 with missing data on weight or WC at baseline or year 3 and 5292 with missing data on covariates. We included 58 961 women in analyses.

### Outcomes

Outcomes included all-cause, cancer, cardiovascular, and other mortality through the end of follow-up February 28, 2023. Annual ascertainment of cause of death in the WHI was based on review of death certificates, regular searches of the National Death Index through 2022, medical records, and other records, such as autopsy. Hospitalization and autopsy records were used whenever possible. A physician in the local clinical center reviewed documents and assigned a diagnosis, which was subject to a central secondary quality control review in some cases.^33^

### Exposures

#### Weight Change

Weight and height were measured at baseline and year 3 by trained personnel using a standard protocol with a balance-beam scale and stadiometer. Height and weight were used to calculate BMI. We calculated weight change between baseline and year 3 and categorized each participant’s change in body weight into 1 of 3 categories: stable weight (change of less than 5% from baseline weight), weight loss (decrease of 5% or more since baseline), and weight gain (increase of 5% or more since baseline).

#### WC Change

WC was measured by trained personnel at baseline and year 3 in centimeters at the natural waist or narrowest part of the torso following a standard protocol. We calculated WC change and grouped participants into 3 categories: stable (change of less than 5% from baseline), loss (decrease of 5% or more since baseline), and gain (increase of 5% or more since baseline).

#### Reported Attempts to Lose Weight

At year 3 follow-up in the WHI-observational study, women were asked whether, in the past 2 years, they had lost 5 or more pounds on purpose or not on purpose at any time. Responses were used to identify women who had intentional weight loss or unintentional weight loss. Among women at the year 3 survey who had measured weight loss or measured WC reduction of 5% or more compared with baseline, we divided these women into 2 groups based on reported intentional or unintentional weight loss (yes or no). If a person responded yes to lost 5 or more pounds on purpose and no to lost 5 or more pounds not on purpose, subsequent measured WC reduction or weight loss were considered intentional. Otherwise, the losses were considered unintentional. Thus, we have measures of 5% WC reduction or 5% weight loss corresponding to intentional or unintentional weight loss. This approach to estimating intentional weight loss or WC reduction has been applied in previous research.^16^

Women who reported intentional weight loss of 5 or more pounds were asked what methods they used to lose weight. We categorized the methods into the most common groups: diet, exercise, a combination of diet and exercise, or other methods. Other methods were relatively less common and included diet pills, surgery, commercial plans, smoking, and other.

#### Covariates

We considered potential confounders at baseline for the multivariate-adjusted models. Continuous variables included age in years, physical activity (metabolic equivalent-hours per week), BMI, and WC. We also considered self-reported race (American Indian or Alaska Native, Asian, Black or African American, Native Hawaiian or Other Pacific Islander, White, and more than 1 race); Hispanic or Latina ethnicity (yes or no); levels of education (high school or less, some college or technical training, college or higher); pack-years of smoking (0, more than 0 to less than 10, 10 to less than 20, 20 to less than 30, 30 to less than 40, and 40 or more); alcohol intake (no use, past use, and current use [less than 1 drink per month, 1 drink per month to less than 1 drink per week, 1 to less than 7 drinks per week, more than 7 drinks per week]), physical activity (metabolic equivalent-hours per week); diabetes (yes or no); cardiovascular disease (yes or no); and prior hormone use (never used, estrogen-alone use, estrogen plus progestin use, mixed).

### Statistical Analysis

We described the distribution of demographic characteristics and potential differences by WC change categories. χ^2^ tests were used to evaluate differences for categorical covariates. For continuous variables, analysis of variance was used. Cox proportional hazards regression models were used to evaluate the associations (hazard ratios [HRs] and 95% CIs) between weight loss, WC reduction, and mortality over 18.6 years of follow-up. Among the group who experienced changes in weight or WC, additional Cox models were used to examine how intentional or unintentional weight loss or WC reduction were associated with mortality outcomes. Weight loss methods were tested for associations with mortality among women who reported intentional weight loss. Finally, we conducted inverse probability weighted (IPW) analysis to determine if missing covariate data might have influenced results. Statistical significance was set at α < .05, and all tests were 2-sided. Data were collected from September 1993 to February 2023 and were analyzed from June to December 2024. SAS version 9.4 (SAS Institute) was used for analysis.

## Results

This study included 58 961 women at baseline (mean [SD] age, 63.3 [7.2] years; mean [SD] BMI, 27.0 [5.6]; mean [SD] WC, 84.1 [13.0] cm). At the end of follow-up, 29 183 women died from all causes (49.5%); 9113 died from cardiovascular disease (15.5%), 5927 died from cancer (10.1%), and 14 143 died from other causes (24.0%). Table 1 presents demographic characteristics by WC change categories. Persons with intentional WC loss had higher baseline BMI and WC than those with stable WC. Differences on all demographic characteristics were statistically significant due to the large sample but seemed of little clinical relevance.

**Table 1.** Baseline Characteristics of Participants by Waist Circumference Change Between Baseline and Year 3^a^

Variables	Participants, No. (%)				
	Overall (n = 58 961)	Waist circumference			
		Stable (n = 34 187)	Loss		Gain (n = 15 521)
			Unintentional weight loss group (n = 5513)	Intentional weight loss group (n = 3740)	
Age, mean (SD)	63.3 (7.2)	63.5 (7.2)	65.2 (7.3)	62.3 (6.9)	62.7 (7.3)
Race					
American Indian or Alaska Native	119 (0.2)	60 (0.2)	18 (0.3)	13 (0.3)	28 (0.2)
Asian	1571 (2.7)	993 (2.9)	177 (3.2)	49 (1.3)	352 (2.3)
Black or African American	4086 (6.9)	2217 (6.5)	444 (8.1)	253 (6.8)	1172 (7.6)
Native Hawaiian or other Pacific Islander	41 (0.1)	23 (0.1)	3 (0.1)	3 (0.1)	12 (0.1)
White	52 494 (89.0)	30 533 (89.3)	4811 (87.3)	3388 (90.6)	13 762 (88.7)
≥1 race	650 (1.1)	361 (1.1)	60 (1.1)	34 (0.9)	195 (1.3)
Hispanic or Latina					
Yes	57 396 (97.3)	33 299 (97.4)	5360 (97.2)	3657 (97.8)	15 080 (97.2)
No	1565 (2.7)	888 (2.6)	153 (2.8)	83 (2.2)	441 (2.8)
Education					
High school diploma	11 547 (19.6)	6594 (19.3)	1220 (22.1)	640 (17.1)	3093 (19.9)
Some college or technical training	21 222 (36.0)	12 223 (35.8)	1989 (36.1)	1364 (36.5)	5646 (36.4)
College degree	14 430 (24.5)	8511 (24.9)	1271 (23.1)	967 (25.9)	3681 (23.7)
Master or higher	11 762 (19.9)	6859 (20.1)	1033 (18.7)	769 (20.6)	3101 (20.0)
Smoking pack-years					
0	31 052 (52.7)	18 159 (53.1)	3001 (54.4)	1868 (49.9)	8024 (51.7)
>0 to <10	12 181 (20.7)	7130 (20.9)	1060 (19.2)	802 (21.4)	3189 (20.5)
10 to <20	5269 (8.9)	3036 (8.9)	490 (8.9)	308 (8.2)	1435 (9.2)
20 to <30	3465 (5.9)	1972 (5.8)	312 (5.7)	245 (6.6)	936 (6.0)
30 to <40	2869 (4.9)	1603 (4.7)	233 (4.2)	236 (6.3)	797 (5.1)
≥40	4125 (7.0)	2287 (6.7)	417 (7.6)	281 (7.5)	1140 (7.3)
Alcohol use					
No use	6148 (10.4)	3581 (10.5)	655 (11.9)	348 (9.3)	1564 (10.1)
Past use	10 166 (17.2)	5690 (16.6)	1030 (18.7)	665 (17.8)	2781 (17.9)
<1 drink/mo	6885 (11.7)	3920 (11.5)	632 (11.5)	454 (12.1)	1879 (12.1)
<1 drink/wk	12 070 (20.5)	6989 (20.4)	1040 (18.9)	777 (20.8)	3264 (21.0)
1 to <7 drinks per wk	15 903 (27.0)	9340 (27.3)	1382 (25.1)	1050 (28.1)	4131 (26.6)
≥7 drinks per wk	7789 (13.2)	4667 (13.7)	774 (14.0)	446 (11.9)	1902 (12.3)
Prior hormone use					
None	22 048 (37.4)	12 791 (37.4)	2247 (40.8)	1375 (36.8)	5635 (36.3)
Estrogen alone	18 228 (30.9)	10 478 (30.6)	1742 (31.6)	1127 (30.1)	4881 (31.4)
Estrogen and progestin	14 736 (25.0)	8585 (25.1)	1161 (21.1)	978 (26.1)	4012 (25.8)
Mixed	3949 (6.7)	2333 (6.8)	363 (6.6)	260 (7.0)	993 (6.4)
Diabetes ever	2695 (4.6)	1511 (4.4)	309 (5.6)	178 (4.8)	697 (4.5)
Cardiovascular disease ever	10 657 (18.1)	6182 (18.1)	1136 (20.6)	627 (16.8)	2712 (17.5)
Physical activity, mean (SD), MET-h/wk	14.1 (14.3)	14.3 (14.4)	12.9 (13.7)	14.0 (14.4)	14.1 (14.2)
BMI, mean (SD)	27.0 (5.6)	26.9 (5.6)	26.8 (5.8)	29.1 (5.8)	26.9 (5.4)
Waist circumference, mean (SD), cm	84.1 (13.0)	84.1 (12.8)	87.2 (14.4)	91.3 (14.2)	81.5 (11.9)

Abbreviations: BMI, body mass index (calculated as weight in kilograms divided by height in meters squared); MET, metabolic equivalent.

^a^ All group differences statistically significant at *P* < .001.

We confirmed that the proportional hazards assumption for Cox models was met. Women who reported intentional weight loss of 5 pounds or more had significantly lower all-cause mortality risk (HR, 0.88; 95% CI, 0.86-0.90), cancer mortality risk (HR, 0.87; 95% CI, 0.82-0.92), cardiovascular disease mortality risk (HR, 0.87; 95% CI, 0.83-0.91), and other mortality risk (HR, 0.87; 95% CI, 0.86-0.92) compared with all other women (Table 2). Women who reported unintentional weight loss had significantly higher all-cause mortality risk (HR, 1.27; 95% CI, 1.24-1.31), cancer mortality risk (HR, 1.25; 95% CI, 1.18-1.32), cardiovascular mortality risk (HR, 1.25; 95% CI, 1.18-1.32), and other mortality risk (HR, 1.25; 95% CI, 1.19-1.31) (Table 2). Among women who reported intentional weight loss, methods of weight loss including diet alone, exercise alone, and diet plus exercise were associated with lower mortality risk in almost all tests, although associations were strongest for combined diet and exercise (all-cause mortality HR, 0.83; 95% CI, 0.80-0.86).

**Table 2.** Association Between Self-Reported Intentional Weight Loss (5 Pounds or More) or Not and Mortality Risk

Outcome	Mortality, HR (95% CI)			
	All-cause	Cancer	Cardiovascular disease	Other
Lose 5 or more pounds intentionally				
No	1 [Reference]	1 [Reference]	1 [Reference]	1 [Reference]
Yes	0.88 (0.86-0.90)	0.87 (0.83-0.92)	0.87 (0.83-0.91)	0.89 (0.86-0.92)
Methods to lose weight				
Diet only	0.90 (0.87-0.93)	0.91 (0.84-0.98)	0.88 (0.82-0.94)	0.91 (0.87-0.96)
Exercise only	0.92 (0.87-0.99)	0.93 (0.80-1.08)	0.82 (0.72-0.94)	0.99 (0.89-1.09)
Diet and exercise	0.83 (0.80-0.86)	0.83 (0.78-0.89)	0.82 (0.77-0.86)	0.84 (0.80-0.88)
Other	0.99 (0.93-1.05)	0.91 (0.79-1.05)	1.06 (0.95-1.18)	0.98 (0.90-1.08)
Lost 5 or more pounds unintentionally				
No	1 [Reference]	1 [Reference]	1 [Reference]	1 [Reference]
Yes	1.27 (1.24-1.31)	1.25 (1.18-1.32)	1.25 (1.19-1.31)	1.30 (1.25-1.35)

Abbreviation: HR, hazard ratio.

 Compared with participants with stable weight, weight gain, overall weight loss, and unintentional weight loss were all associated with significantly greater mortality rates across all groups (Table 3). Intentional weight loss was associated with significantly lower cardiovascular disease mortality (HR, 0.90; 95% CI, 0.81-0.99), and was significantly associated with higher other cause of death outcomes (HR, 1.16; 95% CI, 1.08-1.25) (Table 3).

**Table 3.** Association Between Measured Weight or WC Change (5% or More) From Baseline to Year 3 and Mortality Risk

Outcome	Mortality, HR (95% CI)			
	All-cause	Cancer	Cardiovascular disease	Other
Weight change^a^				
Stable weight	1 [Reference]	1 [Reference]	1 [Reference]	1 [Reference]
Weight gain	1.08 (1.05-1.12)	1.11 (1.04-1.19)	1.11 (1.05-1.17)	1.05 (1.00-1.10)
Weight loss	1.33 (1.29-1.38)	1.24 (1.17-1.33)	1.20 (1.13-1.28)	1.47 (1.40-1.54)
Intentional loss	1.05 (0.99-1.10)	1.02 (0.91-1.14)	0.90 (0.81-0.99)	1.16 (1.08-1.25)
Unintentional loss	1.54 (1.48-1.56)	1.43 (1.30-1.56)	1.40 (1.31-1.50)	1.69 (1.60-1.78)
WC change^b^				
Stable	1 [Reference]	1 [Reference]	1 [Reference]	1 [Reference]
Gain	1.05 (1.02-1.06)	1.06 (1.00-1.12)	1.05 (1.00-1.11)	1.05 (1.01-1.09)
Loss	1.05 (1.01-1.08)	0.99 (0.92-1.06)	0.98 (0.92-1.03)	1.12 (1.07-1.17)
Intentional weight loss	0.91 (0.86-0.95)	0.85 (0.76-0.95)	0.79 (0.72-0.87)	1.01 (0.94-1.08)
Unintentional weight loss	1.14 (1.09-1.18)	1.08 (0.99-1.18)	1.09 (1.02-1.16)	1.18 (1.12-1.25)

Abbreviations: HR, hazard ratio; WC, waist circumference.

^a^ HRs for overall WC or weight loss, vs intentional and unintentional WC or weight loss, were from 2 different models. Results for overall loss were from a model with 3 exposure categories (stable, gain, and loss), and results for intentional and unintentional loss were from a model with 4 exposure categories (stable, gain, intentional, and unintentional).

WC reductions for women with reported intentional weight loss were associated with significantly lower mortality outcomes for all-cause mortality risk (HR, 0.91, HR, 0.86-0.95), cancer mortality risk (HR, 0.85; 95% CI, 0.76-0.95), and cardiovascular disease mortality risk (HR, 0.79; 95% CI, 0.72-0.87) (Table 3). Increased WC was associated with higher all-cause, cancer, cardiovascular, and other mortality. Unintentional WC loss was associated with increased mortality for all-cause, cardiovascular, and other mortality.

The associations between WC change or weight change and risk of overall mortality were not significantly modified by age (younger than 70 years or 70 years or older), baseline BMI (less than 25, 25 to less than 30, and 30 or more) or smoking (never, former, current). We conducted a sensitivity analysis by excluding women with baseline cardiovascular disease or diabetes. Results were unchanged for both weight change and WC. eTable 1 in Supplement 1 shows the total sample counts and counts of events corresponding to Table 2 and Table 3.

Participants with missing data were significantly different from nonmissing cases on study variables (eTable 2 in Supplement 1). However, results of the IPW analysis were identical to the analyses shown in Table 2 and Table 3.

## Discussion

The findings of this study demonstrated that intentionality of weight loss were strongly associated with subsequent mortality in postmenopausal women. Mortality reductions were observed when intentional weight loss was accompanied by reduced WC. Conversely, weight gain and unintentional weight loss were associated with increased mortality risk as expected, and unintentional WC loss was also associated with higher mortality risk. Intentional weight loss with reduced WC was associated with lower all-cause, cancer, and cardiovascular mortality, while intentional weight loss with reduced body weight alone was only associated with lower cardiovascular disease mortality. Unexpectedly, intentional weight loss was linked to higher risk of other mortality.

The differing results in Table 2 and Table 3 may stem from variations in referent groups and exposure measures. Table 2 compared women reporting intentional weight loss to all others, regardless of weight change, while Table 3 examined intentional measured weight loss against a stable weight referent. Table 3 shows significant inverse associations for cardiovascular mortality but not for other causes, with other (noncardiovascular disease and cancer) mortality showing a significant noninverse trend. This likely reflects stronger associations between weight control and cardiovascular health, as weight loss reduces dyslipidemia, blood pressure, and diabetes risk,^34,35,36^ while some cancers and other conditions like infectious or respiratory diseases are less affected by weight loss.

In contrast to weight change, reported intentional weight loss efforts that were coupled with measured WC reductions were associated with lower risk of all-cause, cancer, and cardiovascular mortality. Waist circumference gain and unintentional WC loss were associated with higher mortality risk for all groups.

Reasons for divergent findings between weight and WC changes are not entirely clear. Perhaps older women who attempt to lose weight because they are concerned about adverse health indicators, and successful weight loss efforts of 5% or more are not sufficient to offset those conditions. Mortality weight reduction efforts among older women may have other benefits, such as improved functional ability, reductions in cancer incidence, or improved cardiovascular morbidities.^15,16^

Differences in body composition changes may help explain discrepancies between weight and WC findings. Sarcopenic obesity—obesity combined with age-related muscle mass and strength loss—is increasingly common in older adults.^37^ Intentional weight loss in older women can sometimes lead to muscle loss or weight reduction in extremities rather than abdominal fat loss. This may result from inadequate protein intake or physical activity lacking strength training, both of which contribute to preserving muscle.^38,39^ Strength training in older women has been linked to reduced mortality risk.^40^ Women who successfully reduce WC likely adopt dietary and exercise practices targeting abdominal fat, whereas those losing weight without reducing girth may not. Bowman et al^41^ reported excess mortality in individuals aged 60 to 69 years with high central adiposity and normal or overweight BMI. Visceral adipose tissue, more closely tied to metabolic and cardiovascular risks than subcutaneous fat, plays a key role.^42^ Our findings suggest that combining diet and exercise yields the strongest mortality reduction, although we did not assess specific diets or exercise regimens.

Our findings align with studies that showed intentional weight loss reduces mortality risk.^19,43^ The Kritchevsky et al^19^ meta-analysis included clinical trials of adults with obesity, while our study focused on older women in a population-based setting. Willis et al^43^ examined frequency of intentional weight loss efforts. In contrast, Koster-Rasmussen^5^ found increased all-cause mortality risk for both intentional and unintentional weight loss in patients with overweight and type 2 diabetes. Contradictory findings in prior research^13,19,44^ may stem from differences in anthropometric measures and failure to distinguish between intentional and unintentional weight loss. Our study addressed these distinctions and confirmed that unintentional weight or WC loss likely reflects underlying morbidity.^11^ Our study was strengthened by the large, prospective cohort, long follow-up period, and central adjudication of mortality causes.

### Limitations

This study has limitations. Women reported intentional weight loss efforts but not efforts to reduce WC, so we assumed reductions in WC associated with intentional weight loss were deliberate. Physical activity was assessed at baseline using metabolic equivalents but lacked detail on exercise type. We did not collect information on dietary strategies used in weight loss efforts or distinguish between visceral and subcutaneous fat. Some women reported successfully losing 5 pounds or more but did not achieve measured reductions of 5% or more in weight or WC at year 3. This discrepancy could be due to weight regain,^45,46^ self-report errors, or differences between 5 pounds and 5% reduction (on average, 5% body weight loss corresponded to 7.8 pounds). Weight and WC changes were assessed between baseline and year 3, without accounting for changes afterward. It is possible that women lost weight between baseline and year 3 but regained it later, or vice versa. However, such biases are likely to be nondifferential and would attenuate associations. Baseline covariates did not capture changes after baseline. Most covariates were based on self-report, as was intentionality, which relied on recall. Finally, our results apply to postmenopausal women and may not generalize to other populations.

## Conclusions

We observed that WC reductions that occurred with intentional weight loss efforts were associated with reduced mortality risk in older women across all-cause, cancer, and cardiovascular-related mortality outcomes, but intentional weight loss measured by lower body weight alone was associated with lower mortality risk only from cardiovascular disease. Our findings add to the evidence base that weight may not be the preferred measure for assessing body composition among older women. Our results also suggested that older women with overweight or obesity should not be discouraged from attempting weight loss if they wish to lose weight. Lifestyle changes resulting in reductions in visceral adiposity should be the focus, such as encouraging physical activity that includes strength training to preserve or build muscle mass and dietary changes that promote heart-healthy diets, which may include calorie restriction but also provides adequate protein.^47^

## Notes

Supplement 1. **eTable 1. **Sample Counts and Event Counts Corresponding to Tables 2 and 3 **eTable 2. **Comparison of Baseline Characteristics Between Women Excluded Due to Missing Data and Women in the Final Study Sample

Supplement 2.
Data Sharing Statement
