# Supplementary material for: Intentional Weight Loss, Waist Circumference Reduction, and Mortality Risk Among Postmenopausal Women
Source: JAMA Netw Open. 2025 Mar 6;8(3):e250609. doi: 10.1001/jamanetworkopen.2025.0609 (PMC11886725; doi:10.1001/jamanetworkopen.2025.0609)
Supplement: Supplement 1. — eTable 1. Sample Counts and Event Counts Corresponding to Tables 2 and 3 eTable 2. Comparison of Baseline Characteristics Between Women Excluded Due to Missing Data and Women in the Final Study Sample [file jamanetwopen-e250609-s001.pdf]

Supplemental Online Content

Hendryx M, Manson JE, Ostfeld RJ, et al. Intentional weight loss, waist circumference reduction, and mortality risk among postmenopausal women. *JAMA Netw Open*. 2025;8(3):e250609. doi:10.1001/jamanetworkopen.2025.0609

eTable 1. Sample Counts and Event Counts Corresponding to Tables 2 and 3

eTable 2. Comparison of Baseline Characteristics Between Women Excluded Due to Missing Data and Women in the Final Study Sample

This supplemental material has been provided by the authors to give readers additional information about their work.

© 2025 Hendryx M et al. *JAMA Network Open*.

eTable 1. Sample counts and event counts corresponding to Tables 2 and 3.

Table 2 Counts		Death		Cause of Death			
		No	Yes	Alive	Died from Cancer	Died from CVD	Died from others
	Total N	N	N	N	N	N	N
**Lose 5 or more pounds intentionally**							
**No**	30163	13800	16363	13800	3199	5179	7985
**Yes**	26190	14808	11382	14808	2452	3423	5507
**Don't know**	2608	1170	1438	1170	276	511	651
**Methods to lose weight**							
**NA**	32964	15049	17915	15049	3496	5723	8696
**Diet only**	8071	4216	3855	4216	815	1185	1855
**Exercise only**	1961	1118	843	1118	186	231	426
**Diet and exercise**	13818	8321	5497	8321	1216	1617	2664
**Others**	2147	1074	1073	1074	214	357	502
**Lose 5 or more pounds unintentionally**							
**No**	42390	22870	19520	22870	4063	5991	9466

© 2025 Hendryx M et al. *JAMA Network Open*.

Table 2 Counts	Total N	Death		Cause of Death			
		No	Yes	Alive	Died from Cancer	Died from CVD	Died from others
		N	N	N	N	N	N
**Yes**	13332	5486	7846	5486	1521	2504	3821
**Don't know**	3239	1422	1817	1422	343	618	856

Table 3 Counts	N	All Discovered Death		Cause of Death			
		No	Yes	Alive	Died from Cancer	Died from CVD	Died from others
		N	N	N	N	N	N
**Weight Change**							
**Stable weight**	39516	20192	19324	20192	3842	6117	9365
**Weight gain**	11566	6453	5113	6453	1183	1569	2361
**Weight loss**	7879	3133	4746	3133	902	1427	2417
**Intentional weight loss**	3303	1725	1578	1725	331	430	817
**Unintentional weight loss**	4576	1408	3168	1408	571	997	1600

© 2025 Hendryx M et al. *JAMA Network Open*.

Table 3 Counts		All Discovered Death		Cause of Death			
		No	Yes	Alive	Died from Cancer	Died from CVD	Died from others
	N	N	N	N	N	N	N
**WC Change**							
**Stable**	34187	17378	16809	17378	3416	5313	8080
**WC gain**	15521	8160	7361	8160	1565	2275	3521
**WC loss**	9253	4240	5013	4240	946	1525	2542
**Intentional WC loss**	3740	2050	1690	2050	339	464	887
**Unintentional WC loss**	5513	2190	3323	2190	607	1061	1655

© 2025 Hendryx M et al. *JAMA Network Open*.

eTable 2. Comparison of baseline characteristics between women excluded due to missing data and women in the final study sample. [a]

Variables	Overall N=80638	Women with missing data (N=21677)	Women in the final study sample (N=58961)
**Age**	63.4 ± 7.3	63.5 ± 7.6	63.3 ± 7.2
**Race**			
American Indian or Alaska Native	288 (0.4%)	169 (0.8%)	119 (0.2%)
Asian	2283 (2.8%)	712 (3.3%)	1571 (2.7%)
Native Hawaiian/Other Pacific Islander	54 (0.1%)	13 (0.1%)	41 (0.1%)
Black/African American	6559 (8.1%)	2473 (11.4%)	4086 (6.9%)
White	68703 (85.2%)	16209 (74.8%)	52494 (89.0%)
More than one race	833 (1.0%)	183 (0.8%)	650 (1.1%)
**Hispanic/Latina**			
Hispanic (Yes)	76108 (94.4%)	18712 (86.3%)	57396 (97.3%)
Hispanic (No)	3687 (4.6%)	2122 (9.8%)	1565 (2.7%)
**Education**			
High school diploma	17264 (21.6%)	5717 (27.2%)	11547 (19.6%)

© 2025 Hendryx M et al. *JAMA Network Open*.

Variables	Overall N=80638	Women with missing data (N=21677)	Women in the final study sample (N=58961)
Some college/technical training	29141 (36.4%)	7919 (37.7%)	21222 (36.0%)
College degree	18691 (23.4%)	4261 (20.3%)	14430 (24.5%)
Master or higher	14877 (18.6%)	3115 (14.8%)	11762 (19.9%)
**Smoking pack years**			
0	40726 (52.3%)	9674 (51.1%)	31052 (52.7%)
>0 - <10	15924 (20.4%)	3743 (19.8%)	12181 (20.7%)
10 - <20	6951 (8.9%)	1682 (8.9%)	5269 (8.9%)
20 - <30	4608 (5.9%)	1143 (6.0%)	3465 (5.9%)
30 - <40	3824 (4.9%)	955 (5.0%)	2869 (4.9%)
40 or more	5854 (7.5%)	1729 (9.1%)	4125 (7.0%)
**Alcohol use**			
Non drinker	9036 (11.3%)	2888 (13.7%)	6148 (10.4%)
Past drinker	14900 (18.6%)	4734 (22.5%)	10166 (17.2%)
<1 drink/month	9283 (11.6%)	2398 (11.4%)	6885 (11.7%)
<1 drink/week	16205 (20.2%)	4135 (19.6%)	12070 (20.5%)
1 - <7 drinks per week	20601 (25.7%)	4698 (22.3%)	15903 (27.0%)
7+ drinks per week	10017 (12.5%)	2228 (10.6%)	7789 (13.2%)
**Prior hormone use**			

© 2025 Hendryx M et al. *JAMA Network Open*.

Variables	Overall N=80638	Women with missing data (N=21677)	Women in the final study sample (N=58961)
No use	31743 (39.4%)	9695 (44.7%)	22048 (37.4%)
E alone use	24941 (30.9%)	6713 (31.0%)	18228 (30.9%)
E+P use	18982 (23.5%)	4246 (19.6%)	14736 (25.0%)
Mixed use	4972 (6.2%)	1023 (4.7%)	3949 (6.7%)
**Diabetes ever**	4423 (5.5%)	1728 (8.0%)	2695 (4.6%)
**Cardiovascular disease ever**	14537 (18.5%)	3880 (19.6%)	10657 (18.1%)
**Physical activity (MET-hours/week**	13.8 ± 14.4	12.7 ± 14.6	14.1 ± 14.3
**BMI (mean, kg/m2)**	27.3 ± 5.8	28.2 ± 6.2	27.0 ± 5.6
**Waist circumference, cm**	84.9 ± 13.5	86.9 ± 14.5	84.1 ± 13.0

a. Differences between women with and without missing data were significant at $p<0.001$ for all variables except age, which was significant at $p<0.02$.

© 2025 Hendryx M et al. *JAMA Network Open*.
