# Supplementary material for: Intentional Weight Loss, Waist Circumference Reduction, and Mortality Risk Among Postmenopausal Women
Source: JAMA Netw Open. 2025 Mar 6;8(3):e250609. doi: 10.1001/jamanetworkopen.2025.0609 (PMC11886725; doi:10.1001/jamanetworkopen.2025.0609)
Supplement: Supplement 2. — Data Sharing Statement [file jamanetwopen-e250609-s002.pdf]

Data Sharing Statement

Data
Data available: No

Additional Information
Explanation for why data not available: Data are from the Women's Health Initiative (WHI). Access to data for other researchers is available following WHI procedures for data access.
